# Typographical Errors

**Published:** 1890-02

**Authors:** 


### TYPOGRAPHICAL ERRORS.

We regret extremely the occurrence of such a large number of typographical errors in the January number of the "Journal." Hurry and carelessness in the publishing office, partly due to the removal of the presses from one building to another, was the cause. Corrected page proofs were not corrected in the printing house. Corrections of errors in dates and the more serious ones will be found in the subjoined *Corrigenda :*

Page 21, line 22, read "1887-88" for 1877-78.

Page 35, Fig. 1 in plate read "PM" for AM ; read "DM" for MC.

Page 42, third line from bottom, read "Chicago Stock Yards in 1879 and one in Iowa in 1881."

Page 51, line 27, read "1885" for 1855.

